# Kinematics of male *Eupalaestrus weijenberghi* (Araneae, Theraphosidae) locomotion on different substrates and inclines

**DOI:** 10.7717/peerj.7748

**Published:** 2019-09-26

**Authors:** Valentina Silva-Pereyra, C Gabriel Fábrica, Carlo M. Biancardi, Fernando Pérez-Miles

**Affiliations:** 1 Unidad de Investigación en Biomecánica de la Locomoción Humana, Departamento de Biofísica, Facultad de Medicina, Universidad de la República, Montevideo, Uruguay; 2 Laboratorio de Biomecánica y Análisis del Movimiento, Departamento de Ciencias Biológicas, Centro Universitario Regional Litoral Norte, Universidad de la República, Paysandú, Uruguay; 3 Sección Entomología, Facultad de Ciencias, Universidad de la República, Montevideo, Uruguay

**Keywords:** Tarantulas, Mechanical-work, Gait analysis, Adhesion, Body models

## Abstract

**Background:**

The mechanics and energetics of spider locomotion have not been deeply investigated, despite their importance in the life of a spider. For example, the reproductive success of males of several species is dependent upon their ability to move from one area to another. The aim of this work was to describe gait patterns and analyze the gait parameters of *Eupalaestrus weijenberghi* (Araneae, Theraphosidae) in order to investigate the mechanics of their locomotion and the mechanisms by which they conserve energy while traversing different inclinations and surfaces.

**Methods:**

Tarantulas were collected and marked for kinematic analysis. Free displacements, both level and on an incline, were recorded using glass and Teflon as experimental surfaces. Body segments of the experimental animals were measured, weighed, and their center of mass was experimentally determined. Through reconstruction of the trajectories of the body segments, we were able to estimate their internal and external mechanical work and analyze their gait patterns.

**Results:**

Spiders mainly employed a walk-trot gait. Significant differences between the first two pairs and the second two pairs were detected. No significant differences were detected regarding the different planes or surfaces with respect to duty factor, time lags, stride frequency, and stride length. However, postural changes were observed on slippery surfaces. The mechanical work required for traversing a level plane was lower than expected. In all conditions, the external work, and within it the vertical work, accounted for almost all of the total mechanical work. The internal work was extremely low and did not rise as the gradient increased.

**Discussion:**

Our results support the idea of considering the eight limbs functionally divided into two quadrupeds in series. The anterior was composed of the first two pairs of limbs, which have an explorative and steering purpose and the posterior was more involved in supporting the weight of the body. The mechanical work to move one unit of mass a unit distance is almost constant among the different species tested. However, spiders showed lower values than expected. Minimizing the mechanical work could help to limit metabolic energy expenditure that, in small animals, is relatively very high. However, energy recovery due to inverted pendulum mechanics only accounts for only a small fraction of the energy saved. Adhesive setae present in the tarsal, scopulae, and claw tufts could contribute in different ways during different moments of the step cycle, compensating for part of the energetic cost on gradients which could also help to maintain constant gait parameters.

## Introduction

Movement is one of the key traits affecting the life of most animal species and determining their interactions with the environment, including the search for shelter, food, mates, and the ability to escape from predators (Alexander, 1999). Evolutionary selective pressures have driven animals to display patterns of movement that are physiologically efficient, fast, adjustable, or stable. Locomotion through different environments could condition the morphology and physiology of animals (Dickinson et al., 2000).

The order Araneae are able to move on a wide range of surfaces, with a broad locomotor repertoire that includes the capacity to move backwards and turn on the spot (Spagna & Peattie, 2012; Niederegger, 2013; Zeng & Crews, 2018). Considering the characteristics of the spiders, especially their control systems, they are an excellent model to use in the study on the general features of locomotion (Biancardi et al., 2011). Pendulum mechanics have influenced the evolution of spiders that live hanging from their webs, as well as species that undertake an errant terrestrial lifestyle (Moya-Laraño et al., 2008; Blackledge et al., 2009). However, normal terrestrial walking is comparable to an inverted pendulum, which would imply higher energetic costs of locomotion when compared to suspensory, or truly pendular, locomotion (Wolff, Nentwig & Gorb, 2013). The octopedal locomotion of spiders constitutes an extreme condition in terrestrial locomotion, related to the specialization of body segment groups (tagmosis). Other terrestrial taxa with more locomotory limbs lack such specialized segments (DelaFuente, 1994).

Locomotion has been widely studied in bipedal and quadrupedal vertebrates (e.g.: Cavagna, Heglund & Taylor, 1977; Heglund et al., 1982), in hexapedal arthropods like cockroaches (Full & Tu, 1990; Full & Tu, 1991; Kram, Wong & Full, 1997), and in functional octopedal arthropods like crabs (Blickhan & Full, 1987). More recently, some research has been carried out on arachnids, which has included work examining both functional hexapedal harvestmen (Sensenig & Shultz, 2006) and truly octopedal spiders (Herreid & Full, 1980; Shillington & Peterson, 2002; Biancardi et al., 2011; Spagna, Valdivia & Mohan, 2011; Wang et al., 2011; Spagna & Peattie, 2012; Weihmann, 2013; Booster et al., 2015; Grossi et al., 2016a; Shamble et al., 2017; Wilshin et al., 2018). The mechanical energy needed to move a unit mass for a unit distance (external work) is almost the same for all terrestrial legged species, despite the huge morphological differences in size, shape, skeleton, and the number and position of locomotory limbs. These similarities suggest common design constraints for terrestrial locomotion with respect to energy expenditure (Full & Tu, 1990; Sensenig & Shultz, 2006).

The external mechanical work recorded for the locomotion of *Grammostola anthracina* (C.L. Koch, 1842) (Araneae, Theraphosidae) was lower than in other species (Biancardi et al., 2011). However, the cost of transport (i.e., the metabolic energy expenditure to move a unit mass for a unit distance, CoT) recorded for Theraphosidae was comparable to that of other species of the same mass (Herreid & Full, 1980; Anderson & Prestwich, 1985; Shillington & Peterson, 2002; Grossi et al., 2016a). Therefore, the mechanical efficiency (mechanical work/metabolic cost) should be lower for the Teraphosidae species than other species of comparable mass.

In order to study locomotion of Theraphosidae and other groups of spiders, one must consider the scopulae and claw tufts, which are adhesive devices on their legs. These structures could play an important role in locomotion, both on level ground and while climbing (Niederegger & Gorb, 2006; Foelix, 2011; Spagna & Peattie, 2012; Wolff, Nentwig & Gorb, 2013; Wohlfart et al., 2014; Lapinski, Walther & Tschapka, 2015; Pérez-Miles, Perafán & Santamaría, 2015; Wolff & Gorb, 2015; Pérez-Miles et al., 2017). Most of the species of Mygalomorphae (72%) have adhesive setae and they usually display cursorial lifestyles (Wolff, Nentwig & Gorb, 2013; Pérez-Miles et al., 2017). However, the contribution of these features with respect to climbing is subject to controversy (Pérez-Miles, Perafán & Santamaría, 2015; Pérez-Miles et al., 2017). Pérez-Miles, Perafán & Santamaría (2015) analyzed the role of the adhesive setae of different species to improve locomotion on level ground and at different gradients of incline using both glass and Teflon as substrate for their trials. As expected, they determined that glass contributed to higher levels of friction than Teflon (Roth & Willis, 1952; Pérez-Miles, Perafán & Santamaría, 2015). We hypothesized that both the gait parameters and the mechanical work of locomotion should be affected by adhesion patterns based on these findings. Particularly, we expected duty factors to increase on slippery surfaces and also upward slope (Parsons, Pfau & Wilson, 2008; Weihmann, Brun & Pycroft, 2017).

Experiments were performed on male *Eupalaestrus weijenberghi* (Thorell, 1894) and the same substrates and inclines were used that have been employed in previous works (Pérez-Miles, Perafán & Santamaría, 2015). *E. weijenberghi* is a medium-sized tarantula common in the Pampean biogeographic province. In contrast with other spiders, adult males are larger but lighter than females and have longer legs (Costa, Pérez-Miles & Mignone, 2004). Juveniles and adult females live in burrows that they dig in the soil of meadows. Adult males leave their burrows in search for females after the maturation molt and during the reproductive season. Males walk persistently day and night, and usually when the weather is cloudy and wet. Their adult lifespan is typically only about two months and it is spent in the pursuit of females with little time spent on feeding (Pérez-Miles et al., 2005).

Our objective was to analyze the gait patterns, parameters, mechanics, and energetics of locomotion in this species and how it is affected by changes in substrate inclinations and surfaces. Results are discussed in relation to the biology of this terrestrial arachnid.

## Materials & Methods

### Animals

The experiments were carried out with *E. weijenberghi* males that were collected in March of 2013 (*n* = 12; mass = 5.25 ± 0.63 g; 1.61 ± 0.16 cm; mean ± SD) from Uruguay, Canelones, and Salinas North. Individuals were maintained in glass vials 6.5 cm in diameter by 12 cm high, with soil and water provisions. They were fed weekly with *Blaptica dubia* (Blattodea, Blaberidae). Voucher specimens were deposited in the Arachnological Collection of the Facultad de Ciencias, Universidad de la República, Montevideo, Uruguay (FCE-My).

### Experimental procedure

**Figure 1:**
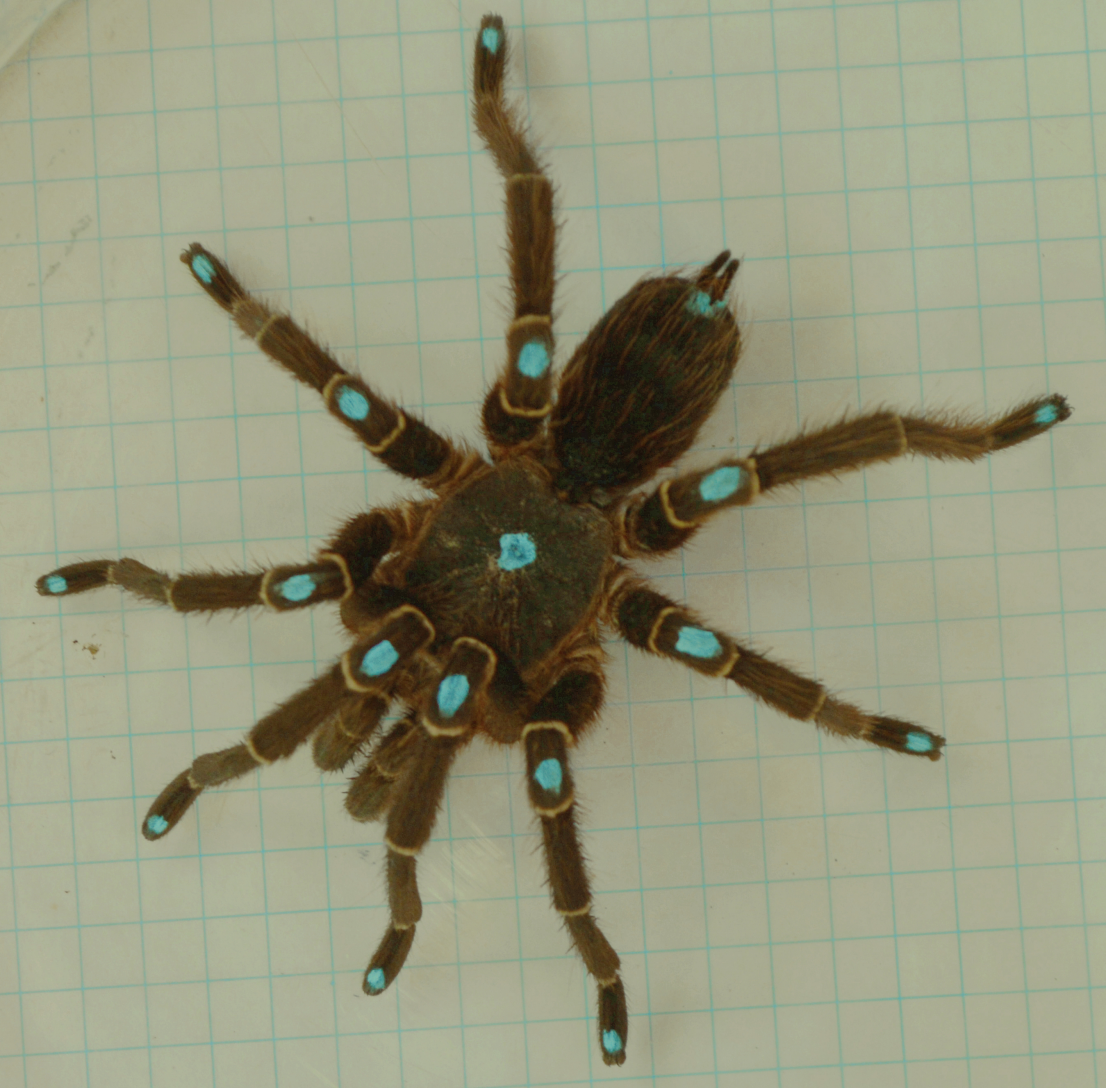
Male *Eupalaestrus weijenberghi*. Light blue marks were used as landmarks.

Free and autonomous displacements of the spiders were recorded during the reproductive season (March–April 2013) on four smooth surfaces: glass and Teflon on level (g0; t0) and glass and Teflon on a 12° incline (g12; t12). The inclination used was selected after considering the average value of the possible locomotion range on Teflon (Pérez-Miles, Perafán & Santamaría, 2015). The locomotion of each individual was recorded using all the experimental conditions in randomized order, with time allowed for rest that spanned at least 48 h between two successive trials. Individuals were marked dorsally, five minutes before the first trial, with water-based, non-toxic ink. Mark positions were on the fovea, the center of the patella, and the tip of the tarsus. These points, together with the light bands in the tibia-metatarsus joint and the points of insertion of each leg on the cephalothorax (coxae), were used as landmarks (Fig. 1). During the trials the temperature was 22.3 °C (±0.6).

**Figure 2:**
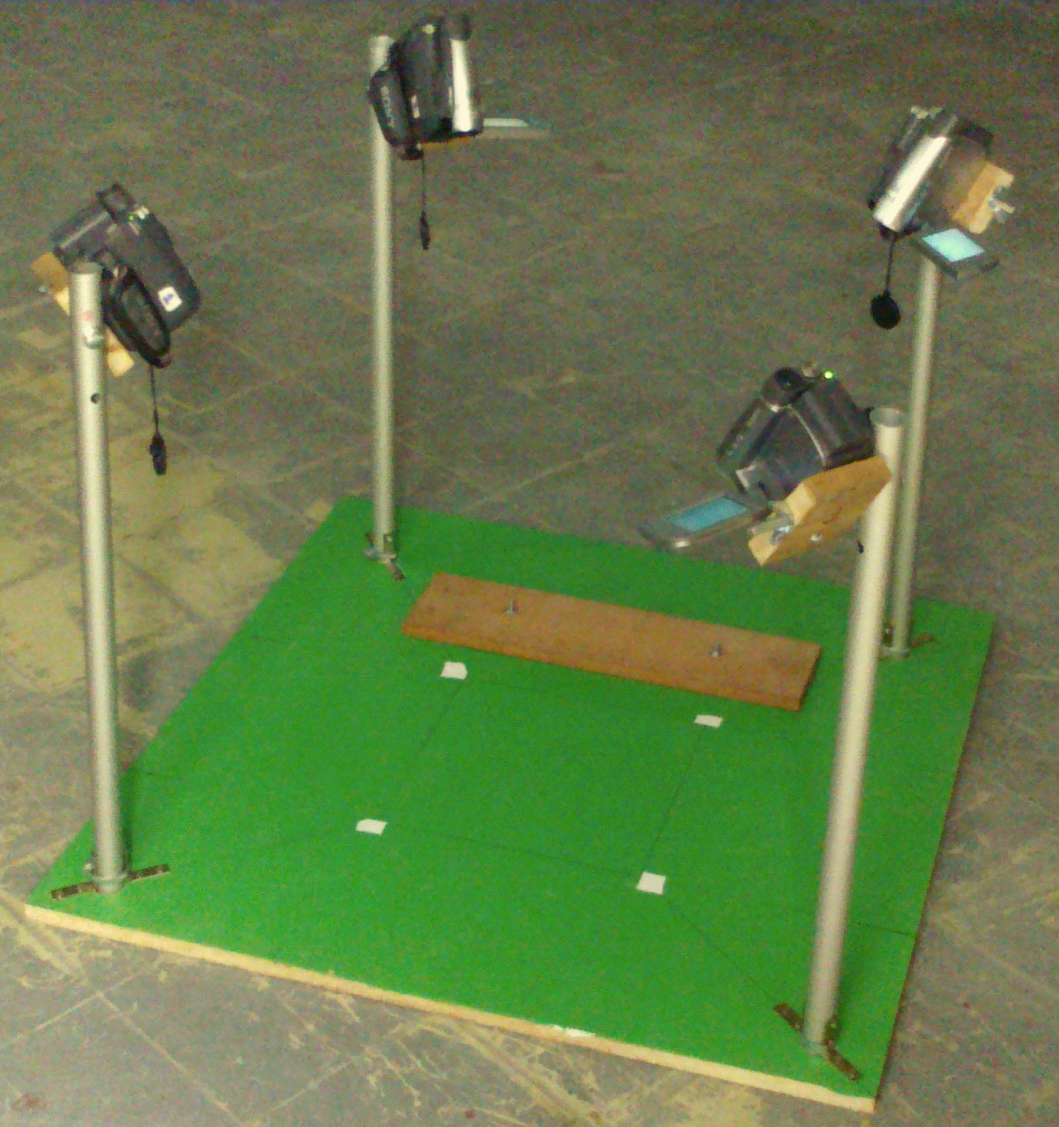
Experimental set-up.

Four fixed video cameras (Sony DCR-H28E) were simultaneously used, within a space of 20 × 32 × 30 cm (Fig. 2). The frame rate was 25 Hz interlaced (50 fps) and we obtained a mean of 50 ± 25 frames per stride; a frequency considered sufficient for this kind of analysis (Ward & Humphreys, 1981; Biancardi et al., 2011). The calibration was done with 17 markers (0.32 cm radius) evenly distributed in three dimensions. The synchronization of the four cameras was obtained by a sound signal and all images were digitized and later used to reconstruct the position of each marker. The measure of error in the calibration system was 0.7% ± 0.2, according to Barros et al. (2006). Orthogonal axes were defined as follows: the direction of *x*-axis was the main displacement direction, the *z*-axis was the height with respect to the ground and the *y*-axis was determined by the right-hand rule (Wu & Cavanagh, 1995).

### Center of mass determination

All of the spiders used for kinematic analysis were sacrificed by means of carbon tetrachloride vapor and fixed in glass tubes with alcohol. The cephalothorax together with the abdomen was separated from the body as well as the following right body segments: coxae and trochanters, femora, patellae jointly with tibiae, metatarsi and tarsi. The mass of each segment was determined with an analytical scale (Radwag AS 310/C2). Lengths and diameters (Table 1) were taken by means of an ocular micrometer (Olympus G15x) with a stereo microscope Olympus SZH. Four measurements of the cephalothorax width at the level of each coxa pair were taken with a calliper. The distance to the fovea and the insertion of each pair of legs was taken from the clypeus on the medial sagittal line (Table 2).

Each segment, apart from the tarsi, was suspended from at least two points and photographed in static equilibrium (Alexander, 1985) with a Nikon D3200 camera. A fine human hair with a knot tied to a hook on one end and the other attached to the segment with removable glue, was used for this purpose.

Digital pictures were analyzed with the program ImageJ 1.49v (Rasband, 1997-2016). A needle of known length was used to calibrate the pictures. In each picture a straight line was drawn as an extension of the hair through the mooring point and the center of the segment. Next, the pictures of the same segment were overlapped. We recorded the length of the segments (lseg) and the distance from the proximal end to the point where the straight lines cross (*L*). The average relative position of the center of mass (*L*_CoM_) of each segment was calculated as: *L*_CoM=_L∕lseg (Table 1).

### Data processing

All of the recorded trials were viewed and visually analyzed. Replicates were selected for kinematic analysis when spiders travelled in straight lines and without ostensible speed changes. After this filter, the sample size was reduced to 5 spiders (*n* = 5, mass = 5.10 g, 0.68 SD).

**Table 1:** Measures and inertial parameter of leg segments (mean ± standard deviation, *n* = 12).

**Segment**	**Leg**	**Diameter (mm)**	**Length (mm)**	Mass (10^−2^ g)	Center of mass (% from proximal end)	Radius of gyration (mm)	Moment of inertia
Femur	I	2.35 ± 0.37	14.12 ± 0.70	71 ± 16	43.8 ± 15.9	4.11	1.20
	II		12.91 ± 0.61	58 ± 12	51.3 ± 15.7	3.77	0.82
	III		11.48 ± 0.63	68 ± 17	53.6 ± 8.8	3.36	0.77
	IV		14.13 ± 0.71	67 ± 28	55.3 ± 12.7	3.76	1.14
Patella-tibia	I	2.40 ± 0.27 2.25 ± 0.44	16.87 ± 0.73	65 ± 12	39.4 ± 5.6	4.90	1.56
	II		15.11 ± 0.56	45 ± 13	29.6 ± 8.2	4.40	0.87
	III		13.59 ± 0.49	43 ± 15	33.0 ± 6.4	3.80	0.68
	IV		18.52 ± 0.59	70 ± 19	38.7 ± 16.2	5.26	2.02
Metatarsus	I	1.41 ± 0.16	10.11 ± 0.58	16 ± 0.4	55.2 ± 16.9	2.93	0.22
	II		9.95 ± 0.54	14 ± 0.5	53.4 ± 12.1	2.40	0.18
	III		11.18 ± 0.32	17 ± 0.6	48.2 ± 17.2	3.25	0.26
	IV		16.96 ± 0.60	36 ± 23	47.4 ± 21.9	4.68	1.11
Tarsus	I			10 ± 0.3			
	II			0.8 ± 0.3			
	III			0.8 ± 0.4			
	IV			10 ± 0.2			

**Table 2:** Measures of cephalothorax and abdomen (mean ± standard deviation, *n* = 12). The mass reporter here correspond to the cephalothorax plus the abdomen.

**Measures of cephalothorax**	
Distance fovea (mm)	10.29 ± 0.45
Distance leg I (mm)	1.58 ± 0.37
Distance leg II (mm)	5.79 ± 0.58
Distance leg III (mm)	11.57 ± 0.88
Distance leg IV (mm)	15.65 ± 1.20
Width I (mm)	11.32 ± 1.73
Width II (mm)	13.99 ± 0.94
Width II (mm)	13.04 ± 1.38
Width IV (mm)	9.60 ± 1.40
Mass (10^−2^ g)	219 ± 23

Two successive step cycles were analyzed in each trial. An image analysis program (Dvideow 6.3, Campinas University) (Figueroa, Leite & Barros, 2003; Barros et al., 2006) was used to synchronize the recordings and to reconstruct the 3D position of each marker. All of the described landmarks were digitized in this way with two exceptions: first, the tibia-metatarsal joints appeared blurred in the trials on Teflon due to reflections. Therefore, it was possible to digitize them only in trials on glass surfaces. Secondly, the *coxae* were obscured because the insertion points of the legs were rarely seen by more than one camera. In this case eight virtual markers were calculated, according to Biancardi et al. (2011), from the fovea position.

A series of Matlab R2012 (MathWorks, Inc., Natick, MA, USA) routines were built to manage and process the kinematics data. Spatial coordinates of the markers were filtered with a low-pass Butterworth of order 3 and a cut frequency of 6 Hz.

**Figure 3:**
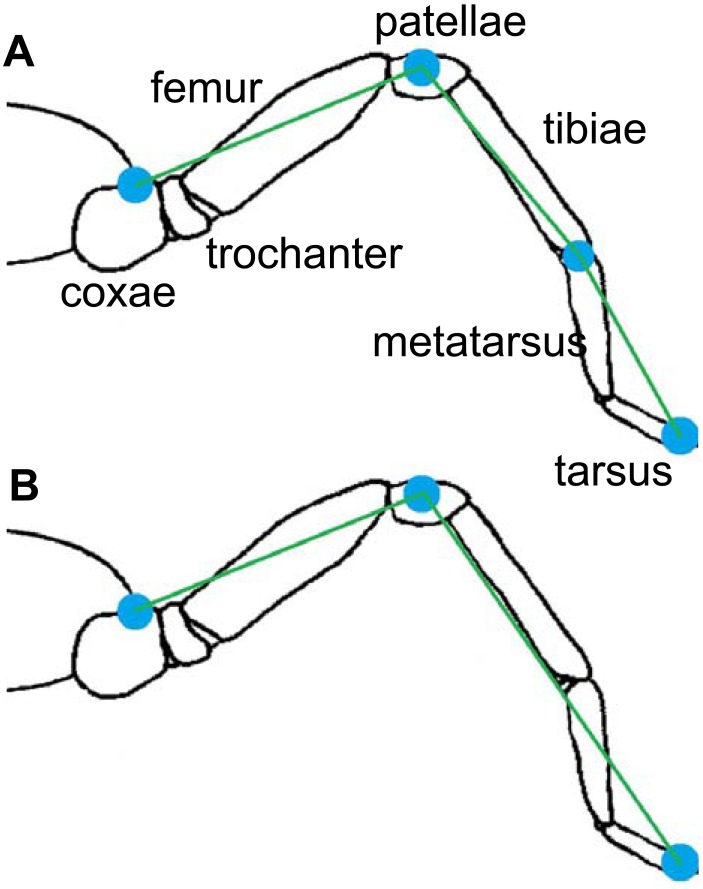
Models of rigid segments of the body of spiders. (A) was used for glass trials and (B) for Teflon.

Two multi-segment body models for the spiders were used (Fig. 3). The cephalothorax segment was delimited by the eight coxae, while the leg segments were delimited by a pair of the described landmarks. The first (model A) considers three segments per leg (femur, patella-tibia, tarsus-metatarsus), while the second (model B) includes only two leg segment groupings (femur, and other distal segments together). In both models, the coxa and trochanter were considered together with the cephalothorax and abdomen. Model A was used for analyzing the trials on the glass surface, while model B was used for the analysis of both surfaces (Teflon and glass). The radii of gyration of the segments (Table 1) were calculated by assuming a cylinder shape for the segments (Biancardi et al., 2011). The 3-D position and respective masses of each *L*_CoM_ with the spatial position of the fovea were used to obtain the frame-by-frame 3-D position of the total body center of mass (*b*_CoM_).

The trajectories of *b*_CoM_ were used to calculate mechanical work measurements and mechanical energies following procedures outlined by Willems, Cavagna & Heglund (1995). The internal work (*W*_INT_) is the increase in kinetic energy of the leg segments arising from their speed change with respect to the *b*_CoM_. The external work (*W*_EXT_) is the increase in kinetic energy of the *b*_CoM_ with respect to the environment. *W*_EXT_ was obtained by summing up the positive increments of total energy (*E*_TOT_) with respect to time: *E*_TOT_ = *E*_POT_ + *E*_KIN.x_ + *E*_KIN.y_ + *E*_KIN.z_, where *E*_pot_ is the potential energy of the *b*_CoM_. *E*_KIN.x_, *E*_KIN.y_, and *E*_KIN.z_ are the forward, lateral, and vertical components of the kinetic energy included in *b*_CoM_, respectively. Vertical and horizontal work (*W*_V_, *W*_H_) components of *W*_EXT_ were obtained by summing up the positive increments of vertical energy (*E*_POT_ + *E*_KIN.z_) and by summing up the positive increments of forward and lateral energy (*E*_KIN.x_+ *E*_KIN.y_), respectively. The total mechanical work (*W*_TOT_) was computed as the sum of the *W*_INT_ and *W*_EXT_ (Cavagna & Kaneko, 1977). Mechanical work was expressed as the mechanical cost of the transport per kilogram of body mass and per unit of distance (i.e., J Kg^−1^m^−1^).

For ideal walking gaits, *E*_POT_ and *E*_KIN_ changes are out of phase, while for bouncing gaits they are in phase. Percentage congruity (% Cong), which is the proportion of a stride cycle during which the two energies change in the same direction, was computed according to Ahn, Furrow & Biewener (2004). The ability of the body to save mechanical energy through the interchange between *E*_POT_ and *E*_KIN_ (energy recovery) was calculated according to Cavagna, Thys & Zamboni (1976).

Different multi-segmented models could affect the determination of the position of the *b*_CoM_ and the estimations of the mechanical work (Pavei et al., 2017). However, the effect of different numbers of limb segments on the *b*_CoM_ trajectory (*W*_EXT_) was almost negligible, especially in cases where the limb masses/body mass ratio is small (Table 1). *W*_INT_ could be more greatly affected by the number of segments (Biancardi et al., 2011).

There were additional parameters estimated from the analysis of the trajectory of *b*_COM_. The mean height of vertical displacement of the *b*_COM_(H_COM_) was one these parameters. Speed was determined by the displacement of the *b*_COM_ in the horizontal plane/time. The relative speed to body length was calculated using measurements for the body length (the sum of cephalothorax and abdomen length). The stride length *L*_S_ was defined as horizontal *b*_COM_ displacement during one stride, while the relative stride length was defined as *L*_SR_ = *L*_S_∕*H*_COM_. We also estimated the stride frequency (*F*_S_) and the duty factor (*D*_*F*_ is the ratio between the duration of a foot contact interval and the stride duration).

In order to perform the gait analysis the spider was considered to be composed of two successive quadrupeds in series (Root, 1985; Biancardi et al., 2011). The first being L1-R1-L2-R2 and the second L3-R3-L4-R4 (where L and R indicate left and right and the numbering continues sequentially from the first fore-pair of legs). The gait diagram of each quadruped was compared with the theoretical quadrupedal gait diagrams (Hildebrand, 1966; Hildebrand, 1977).

**Figure 4:**
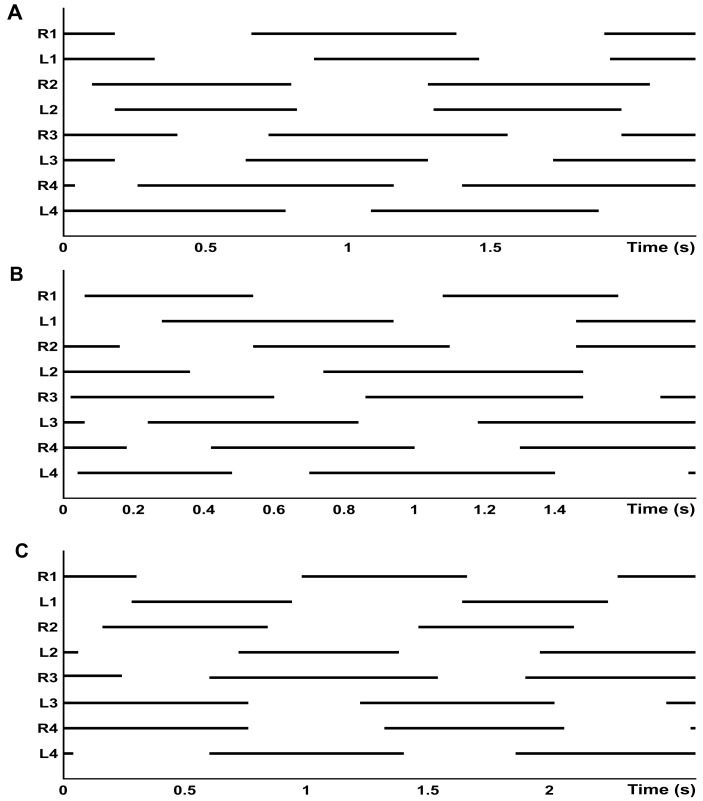
Gait diagram of footfall pattern. (A) Walk-bound in legs I and II (R1 to L2) and diagonal walk in legs III and IV (R3 to L4), recorded on g0. (B) Lateral walk in all legs, recorded on t0. (C) Walk-trot in all legs, recorded on g12.

Stride coordination was evaluated by the antero-posterior sequence method (Abourachid, 2003) and considered the sequence of footfalls as pictured in the gait diagram (Fig. 4). The parameters included the time lag between two contralateral feet footfalls measuring temporal coordination within each pair of fore (F) or hind (H) limbs: *F*_l_1 (L1-R1), *H*_l_1 (L2-R2), *F*_l_2 (L3-R3) and *H*_l_2 (L4-R4). They also included the time lag between fore and hind feet footfalls on the same side, for leg pair I-II (P_l_1) and for leg pair III-IV (P_l_2), all of which were expressed as a percentage of the cycle duration.

All statistical analyses were done using the PAST 3.12 software package (Hammer, Harper & Ryan, 2001). To compare the variables at different experimental conditions, we used a two-way ANOVA statistical analysis for paired values, with the substrate inclinations and surfaces acting as independent factors. The results from the two body models and temporal gait parameters have been compared by *t*-test or univariate ANOVA with a Bonferroni *post-hoc* test. The linear Pearson *r* coefficient was computed to test the correlation between speed and stride frequency. In all cases the critical *p*-value was set to 0.05.

To analyze footfall patterns, we performed two principal components analyses. One included all eight legs and the other considered the tarantulas as two independent quadrupeds with the first set including pairs I and II and the second including pairs III and IV.

## Results

A total of 40 strides were analyzed, with 10 strides for each experimental condition (g0; g12; t0; t12). All of the raw data are given in a Supplemental File.

### Speed and gait measurements

**Figure 5:**
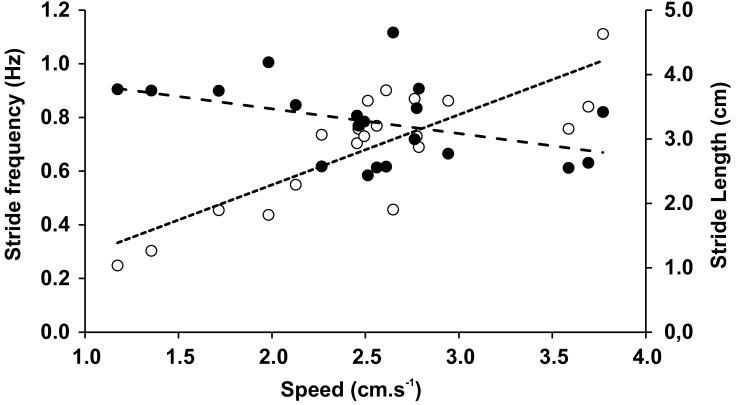
Stride frequency (open circles) and stride length (full circles) on function of speed. The dotted lines are the linear adjustments.

The mean speed recorded during the trials was 2.53 cm × s^−1^ (0.68 SD), with no significant differences between speeds when trials included varied inclinations or substrates. In Tables 3 and 4 we have shown averages that were measured for both the different inclinations and the different substrates with associated statistical comparisons, respectively. Within the observed range of velocities, the stride length (*L*_S_) was almost constant (*r* =  − 0.419, *p* = 0.065), while the stride frequency (*F*_S_) significantly increased with speed (*r* = 0.821, *p* = 0.000009) (Fig. 5). The duty factor was always higher than 0.5 for walking gaits and did not show significant differences between different conditions. The same trends could be observed for stride length, stride frequency, and relative stride length (Tables 3 and 4). The height of the center of mass (H_CoM_) was the only variable affected by the interaction of experimental conditions (*F* = 28.14, *p* = 0.006), caused by a change of the limb posture on Teflon relative to the glass substrate, ranging from a more upright position on level surfaces to a more sprawled one on gradients (Table 3).

**Table 3:** Results obtained at all experimental condition (mean ± standard deviation, *n* = 5).

**Variable**	**Glass 0°**	**Teflon 0°**	**Glass 12°**	**Teflon 12°**
Speed (cm s^−1^)	2.55 ± 0.90	2.80 ± 0.62	2.61 ± 0.59	2.17 ± 0.63
Relative speed (*B*_*l*_*s*^−1^)	1.11 ± 0.38	1.23 ± 0.29	1.03 ± 0.37	0.94 ± 0.25
Duty Factor (*D*_*F*_)	0.61 ± 0.01	0.58 ± 0.04	0.60 ± 0.05	0.59 ± 0.04
*L*_*S*_ (cm)	3.04 ± 0.68	3.53 ± 0.69	3.17 ± 0.68	3.31 ± 0.51
*L*_SR_	2.28 ± 0.56	2.09 ± 0.44	2.25 ± 0.23	2.43 ± 0.28
*F*_*S*_ (s^−1^)	0.71 ± 0.27	0.74 ± 0.26	0.69 ± 0.14	0.62 ± 0.23
*H*_CoM_ (cm)	1.34 ± 0.06	1.70 ± 0.19	1.41 ± 0.13	1.34 ± 0.11
*W*_EXT_ mJ kg^−1^ m^−1^)	568 ± 98	1,202 ± 1,038	2,135 ± 128	2,578 ± 721
*W*_INT_ (mJ kg^−1^ m^−1^)	1.1 ± 0.6	2.6 ± 0.8	0.9 ± 0.3	2.4 ± 1.9
*W*_TOT_ (mJ kg^−1^ m^−1^)	569 ± 98	1,205 ± 1,038	2,136 ± 129	2,578 ± 721
*W*_V_ (mJ kg^−1^ m^−1^)	563 ± 97	1,205 ± 1,053	2,137 ± 129	2,574 ± 719
*W*_*H*_ (mJ kg^−1^ m^−1^)	45 ± 16	95 ± 40	43 ± 9	53 ± 25
Recovery (%)	6.9 ± 3.8	10.5 ± 9.3	2.1 ± 0.7	1.9 ± 0.7
Percentage congruity (%)	53.05 ± 7.89	47.74 ± 1.95		

**Table 4:** Results of the two-way ANOVA.

**Variable**	**Gradient effect**	**Substrate effect**	**Gradient x Substrate**
Speed	*F* = 0.984; *df* = 1; *p* = 0.377	*F* = 0.146; *df* = 1; *p* = 0.722	*F* = 1.504; *df* = 1; *p* = 0.287
Duty factor	*F* = 0.080; *df* = 1; *p* = 0.791	*F* = 1.720; *df* = 1; *p* = 0.260	*F* = 0.460; *df* = 1; *p* = 0.535
Stride length	*F* = 0.046; *df* = 1; *p* = 0.841	*F* = 3.166; *df* = 1; *p* = 0.150	*F* = 0.980; *df* = 1; *p* = 0.378
Relative stride length	*F* = 1.000; *df* = 1; *p* = 0.374	*F* = 0.001; *df* = 1; *p* = 0.977	*F* = 3.380; *df* = 1; *p* = 0.140
Stride frequency	*F* = 1.463; *df* = 1; *p* = 0.293	*F* = 0.138; *df* = 1; *p* = 0.729	*F* = 0.281; *df* = 1; *p* = 0.624
b_**COM**_ height	*F* = 4.108; *df* = 1; *p* = 0.113	**F** = **24.874**; *df* = 1; *p* = 0.008	*F* = 28.139; *df* = 1; *p* = 0.006
External work	*F* = 19.260; *df* = 1; *p* = 0.012	*F* = 4.645; *df* = 1; *p* = 0.097	*F* = 0.096; *df* = 1; *p* = 0.772
Internal work	*F* = 9.041; *df* = 1; *p* = 0.040	**F = 33.972; *df* = 1; *p* = 0.004**	*F* = 1.677; *df* = 1; *p* = 0.265
Total work	*F* = 19.204; *df* = 1; *p* = 0.012	*F* = 4.672; *df* = 1; *p* = 0.097	*F* = 0.096; *df* = 1; *p* = 0.773
Vertical work	*F* = 19.191; *df* = 1; *p* = 0.012	*F* = 4.506; *df* = 1; *p* = 0.101	*F* = 0.108; *df* = 1; *p* = 0.759
Horizontal work	*F* = 5.642; *df* = 1; *p* = 0.076	*F* = 17.760; *df* = 1; *p* = 0.014	*F* = 4.090; *df* = 1; *p* = 0.113
Recovery	*F* = 5.622; *df* = 1; *p* = 0.077	*F* = 2.038; *df* = 1; *p* = 0.227	*F* = 1.656; *df* = 1; *p* = 0.268

**Notes.**

The bold text indicates results with significant differences, *p* < 0.05.

**Table 5:** (A) Time lags and (B) duty factors of each leg pair for different gaits (mean ± standard deviation, *n* = 5).

**A**	*F*_*l*_1	*H*_*l*_1	*P*_*l*_1	*F*_*l*_2	*H*_*l*_2	*P*_*l*_2
Diagonal	0.38 ± 0.13	0.51 ± 0.19	0.45 ± 0.16	0.31 ± 0.15	0.54 ± 0.14	0.45 ± 0.07
Lateral	0.40 ± 0.11	0.72 ± 0.54	0.72 ± 0.37	0.38 ± 0.18	0.55 ± 0.15	0.57 ± 0.12
Trot	0.42 ± 0.15	0.51 ± 0.09	0.49 ± 0.14	0.44 ± 0.09	0.51 ± 0.04	0.43 ± 0.08
Bound	0.14 ± 0.08	0.23 ± 0.21	0.44 ± 0.08			

B	*D*_FFl_1	*D*_FHl_1	*D*_FPl_1	*D*_FFl_2	*D*_FHl_2	*D*_FPl_2
Diagonal	0.53 ± 0.06	0.61 ± 0.09	0.56 ± 0.08	0.68 ± 0.08	0.65 ± 0.07	0.67 ± 0.06
Lateral	0.49 ± 0.04	0.58 ± 0.10	0.52 ± 0.08	0.66 ± 0.06	0.71 ± 0.04	0.69 ± 0.04
Trot	0.50 ± 0.02	0.51 ± 0.04	0.52 ± 0.04	0.63 ± 0.07	0.65 ± 0.04	0.64 ± 0.04
Bound	0.53 ± 0.04	0.60 ± 0.04	0.59 ± 0.04			

Gait diagrams show four different gaits, sometimes determined by the composition of different patterns between the two quadrupeds (Fig. 4). Walk-bound was only observed in the anterior quadruped and was characterized by the simultaneous contact of the contralateral feet with a phase shift of about 50% between leg pairs I and II (similar to a quadrupedal bound, without an aerial phase, as seen in Fig. 4A). For the walk-trot gait the odd limbs of the right side (I and III) move simultaneously with the even limbs of the left side (II and IV), like in a quadrupedal trot, again without an aerial phase. Diagonal and lateral walk patterns are equivalent to their quadrupedal homonym (Hildebrand, 1966; Abourachid, 2003). The time lags and *D*_*F*_ of each leg pair for different gaits are given in Table 5. The overall *D*_*F*_ of the four leg pairs resulted in significantly different values (*F* = 35.105, *d*.*f*. = 3, *p* < 0.0001). However, between the two pairs of the anterior quadruped (I and II), and between the two pairs of the posterior quadruped (III and IV), the multiple comparison methods resulted in non-significant differences. Bonferroni comparisons of DFF1-DFH1 and DFF2-DFH2 resulted in p-values of 0.238 and 1.000, respectively. All other comparisons gave significant results including Bonferroni comparisons of DFF1-DFF2 and DFH1-DFH2, with significance tests for both with a result of *p* < 0.0001. The pooled *D*_*F*_ of the two quadrupeds again highlighted significant differences (DFP1-DFP2, *t* =  − 9.799, d.f. = 78, *p* < 0.0001).

**Figure 6:**
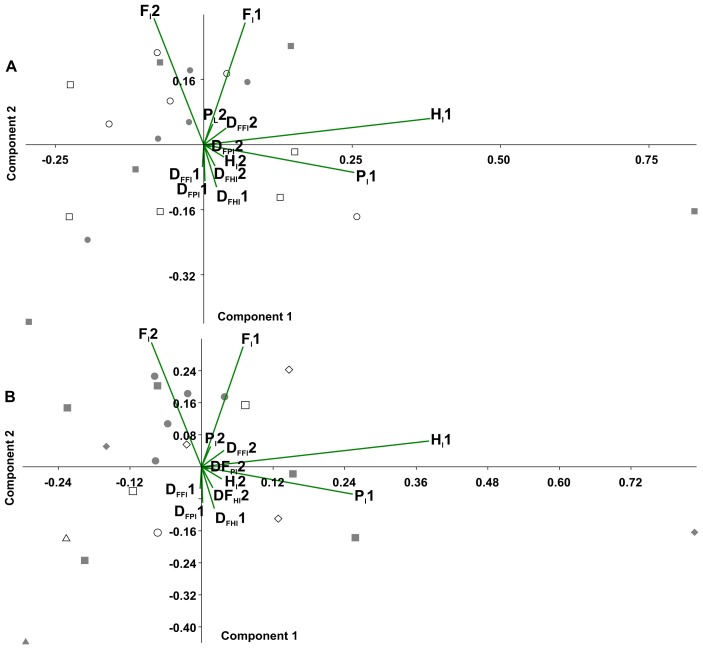
Principal component analysis of temporal variables. Time lag of the pair legs I (*F*_*l*_1), of pair legs II (H_l_1), pair III (*F*_*l*_2) and IV (*H*_*l*_2); time lag of ipsilateral legs, pair I–II (*P*_*l*_1), and between pair III-IV (P_l_2); the duty factor of pair of legs I (*D*_*FFl*_1), pair II (D_FHl_1), III (*D*_*FFl*_2) and IV (*D*_*FHl*_2), ipsilateral of the pairs I–II (*D*_*FPl*_1), and the III–IV (D_FPl_2). (A) Experimental condition (full squares—g0, open circles—g12, full circles—t0, open squares—t12). (B) Gait patterns quadrupeds (full triangle–bound diagonal, open triangle–bound trot, full circles–trot trot, full squares–diagonal diagonal, full diamonds–lateral lateral, open squares–trot diagonal, open diamonds–trot lateral, open circle–diagonal trot).

In the principal component analysis considering the octopod (Fig. 6), PC1 explained the 36.8% of the variance, with the highest loadings of *H*_l_1 (0.80) and *P*_l_1 (0.53). PC2 explained the 21.8% of the variance, with the highest loadings of *F*_l_2 equalling 0.65 and *F*_l_1 reaching 0.63. No significant associations were found between the experimental conditions and the variables (duty factors and time lags) (Fig. 6A). However, the time lags of pairs I and II (*F*_l_1, *H*_l_1, *P*_l_1) tended to lean toward the positive values of PC1. Duty factors associated with these time lags were placed toward the negative values of PC2. Conversely, posterior leg pairs did not show similar tendencies. Temporal variables distinguished the two types of gaits (Fig. 6B). Walk-trot gaits using four leg pairs were observed in the negative quadrant of PC1 and positive quadrant of PC2 while walk-bound gait patterns as seen in pairs I and II were observed in the negative quadrants of PC1 and PC2.

**Figure 7:**
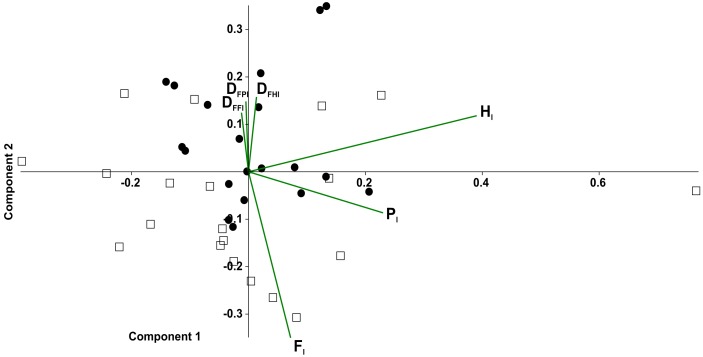
Principal component analysis of temporal variables considering the tarantulas as two quadrupeds. Time lag of fore legs (*F*_*l*_), of hind legs (*H*_*l*_); ipsilateral (*P*_*l*_); and duty factor of the fore legs (*D*_*FFl*_), hind legs (*D*_*FHl*_) and ipsilateral (*D*_*FPl*_). Gait of pair legs I and II (open squares) and pair legs III and IV (full circles).

In the principal component analysis considering quadrupeds (Fig. 7), PC1 explained 35.2% of the variance, with the highest loadings being H_l_(0.85) and P_l_(0.50). PC2 explained the 25.6% of the variance, with *F*_l_ (0.77) being the highest loading, followed by duty factors. The anterior quadruped showed lower values than the posterior quadruped of PC2; consequently a *t*-test was performed to test the differences between these components. Significant differences were found between the anterior and posterior quadrupeds (*t* =  − 3.02, *p* = 0.01).

### Mechanical work

External work (*W*_EXT_) accounted for the largest part of the total mechanical work (*W*_TOT_), while the internal work component only contributed to less than 1% of *W*_TOT_ (Tables 3 and 4). The increase of the gravitational potential energy due to climbing explains the significant differences between *W*_V_, *W*_EXT,_ and *W*_TOT_ detected on gradients. The internal work (*W*_INT_) was 10% to 20% higher on level surfaces than on gradients, regardless of the type of surface tested (*F* = 9.041, *p* = 0.040). The horizontal work (*W*_H_) was significantly higher on Teflon than glass (*F* = 17.760, *p* = 0.014). The % Cong produced intermediate values (mean 51.7%) between vaulting and bouncing gaits. Recovery was lower on gradients than on level surfaces, but calculated differences were not significant (Tables 3 and 4).

The two models produced different values for internal work, which was generally overestimated by the simplified model B (31%). However, the differences were not statistically significant (*W*_INT_: *t* = 0.775, *d*.*f*. = 8, one-tail *p* = 0.230). Model B produced a slight overestimation (1.46%) for vertical work, and therefore also external work, with no significant effects on the variable means (*W*_V_ and *W*_EXT_: *t* = 0.133, d.f. = 8, one-tail *p* = 0.449). Due to the small contribution of internal work, the effect on the total work was similar to the latter (*W*_TOT_: *t* = 0.136, d.f. = 8, one-tail *p* = 0.447). Horizontal work and recovery were not affected and the mean differences between the two were less than 0.5%, while the *p*-values of the *t*-test were nearly one.

## Discussion

### Gait pattern

Speed changes made by legged animals can be obtained by adjusting the stride frequency and stride length (Cavagna et al., 1988). In researching ants, Zollikofer (1994) hypothesized that long legged arthropods would employ longer strides to go faster. Other authors pointed at *F*_S_ as the main determinant of speed changes in Coleoptera, Carabidae, and cockroaches (Evans, 1977; Ting, Blickhan & Full, 1994). In *E. weijenberghi*, we identified the *F*_S_ values to be the main determinants of speed changes, at least within the limited range studied here. This result agrees with previous findings in other tarantulas (Anderson & Prestwich, 1985; Booster et al., 2015). However, in females of *G. anthracina, L*_S_ gradually increased within a speed range comparable to that observed in *E. weijenberghi* (Biancardi et al., 2011). In general, animals with exoskeletons are forced to modify the *F*_S_ to change speed. The hardness of the skeletal components makes it difficult to modify *L*_S_ through the participation of other body parts, which also happens in animals with endoskeletons (Griffin, Main & Farley, 2004). Considering that the *F*_S_ is related to muscular work (Heglund & Taylor, 1988) and limited by the muscular physiology (Alexander, 2003), higher maximum speeds are achieved by spider species (or genders, in the case of sexual dimorphism) with longer legs (Grossi & Canals, 2015; Grossi et al., 2016a).

*E. weijenberghi*, did not significantly change gait, speed, *F*_S_ or *D*_*F*_ while moving on a slope. Other species will adapt at least a subset of those parameters on an incline (Gabaldón, Nelson & Roberts, 2004; Seidl & Wehner, 2008; Andrada et al., 2013; Birn-Jeffery & Higham, 2014; Wöhrl, Reinhardt & Blickhan, 2017). The presence of leg adhesive setae (claw tufts and scopulae) could increase the friction between the foot and the substrate, consequently reducing the necessity of extra muscular work on an incline (Pérez-Miles, Perafán & Santamaría, 2015).

**Figure 8:**
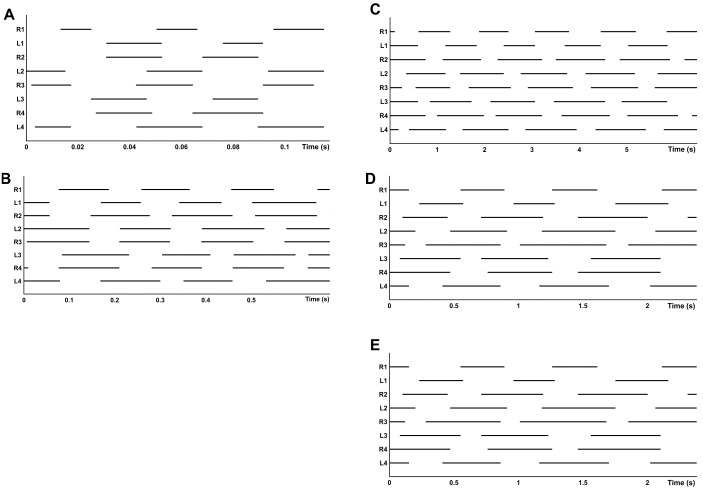
Gait diagram of different species. (A) *Hololena adnexa* (Araneae, Agelenidae) modified from Spagna & Peattie (2012); (B) *Hadrurus arizonensis* (Scorpiones, Iuridae) modified from Root (1985), and (C–E) *G. anthracina* at three speeds modified from Biancardi et al. (2011).

Alternate tripodal and quadrupedal patterns, such as the walk-trot, have already been described in crabs (Blickhan & Full, 1987), cockroaches running at low speed (Ting, Blickhan & Full, 1994), a Caraboctonidae scorpion, an agelenidae spider (Spagna & Peattie, 2012; Figs. 8A–8B), and tarantulas at different velocities (Biancardi et al., 2011). For *G. anthracina,* several gaits were observed, including the walk-trot combined with a lateral walk at low speed, a diagonal walk at medium speed, and walk-trot combined with diagonal walk at high speed (Figs. 8C–8E). Walk-like and trot-like gaits are broadly defined in the symmetrical gait category (Hildebrand, 1966; Abourachid, 2003). An asymmetrical walk-bound gait has also been infrequently observed but only for the anterior quadruped (pairs I and II). All of these gaits agree with the expected time-lag values proposed by Abourachid (2003).

Rapid and intermittent movements were reported, as is commonly observed in theraphosids (Shillington & Peterson, 2002; Grossi et al., 2016b), but the sequences we selected were straight trajectories and at nearly constant speed. Our results agree with those of Biancardi et al. (2011), reaching the conclusion that spider locomotion patterns are complex and do not appear to show rigid neural control. This plasticity enables rapid corrections of locomotion patterns on irregular terrains.

According to the PCA results, the observed patterns of locomotion were characterized by variations of time lags of pairs I, II, and III versus the variations of duty factors. Pair I has both mobility and exploratory functions (Anderson & Prestwich, 1985; Blickhan & Barth, 1985; Foelix, 2011). The exploratory function implies less time of contact with the substrate and this could partially explain the differences in *D*_*F*_ values between the forelimbs and hind limbs.

In mammals, the fore limbs support a great proportion of the total weight due to the weight of the head which produces a higher *D*_*F*_ for the fore limbs versus the posterior limbs (Maes et al., 2008). Our results show a more restricted pattern in the posterior quadruped probably due to the role of pairs III and IV in supporting the body weight of the tarantula. Furthermore, center of mass of the tarantula’s body is located between the insertion of legs III and IV, just behind the fovea (Biancardi et al., 2011). Anterior legs show more versatility, which can be attributed to their involvement in exploratory functions and driving displacements. Traditionally, *D*_*F*_ was the parameter used to analyze locomotory patterns (Farley, Glasheen & McMahon, 1993; Alexander, 2003), while the study of the anterior-posterior sequence is a more recent development (Abourachid, 2003). The principal component analysis provided evidence that both approaches were important to understand gait coordination.

### Mechanical work and efficiency

In different species that have been studied, such as cockroaches (Full & Tu, 1990; Full & Tu, 1991; Kram, Wong & Full, 1997; Weihmann, Brun & Pycroft, 2017), crabs (Blickhan & Full, 1987), quadruped mammals (Heglund et al., 1982), and bipeds (Saibene & Minetti, 2003) mechanical work is quite constant and independent from body mass, body shape, number of legs, or skeletal type (Full & Tu, 1990). Tarantulas seem to be the exception to this rule with the external work in *E. weijenberghi* being about half of that reported for other species, which is in agreement with the observation of Biancardi et al. (2011) for *G. anthracina*. Possibly the hydraulic system of arachnids, or elastic mechanisms present in the distal joints of tarantulas, are involved in the extension of legs and related to this difference (Sensenig & Shultz, 2003).

Females of the *E. weijenberghi* species do not move more than 40 cm away from the entrance of their respective burrows (Álvarez, Perafán & Pérez-Miles, 2006), while adult males walk incessantly in order to search for females (Pérez-Miles et al., 2005). The low magnitude of mechanical energy involved in locomotion would facilitate long movements by males without the need for feeding (Pérez-Miles et al., 2005). Indeed, there is a relationship between the mechanical work of locomotion and the metabolic energy expenditure. A low metabolic cost of transport is one of the characteristics associated with the lifestyle of wandering male spider (Grossi et al., 2016a). However, the low cost determined here is an absolute value, influenced by the low body mass of wandering males with respect to females. When comparing the per unit body mass, the cost of transport of smaller individuals is higher than that of larger ones and therefore their mechanical efficiency is lower. Nevertheless, the optimization of cost and efficiency is not a unique determinant of locomotion, and in some cases, when the cardiovascular or respiratory system are not performing well, inefficient yet economic (in absolute terms) locomotion patterns may be preferred (e.g., Bona et al., 2017).

The increment of *W*_EXT_ during locomotion on a gradient was caused by the additional *W*_V_, and there were proportionally similar observations made in humans (Minetti, Ardigo & Saibene, 1993; Gottschall & Kram, 2006). This increment was slightly lower on Teflon than on glass. On Teflon the *W*_H_ was lower, probably due to the lower level of adhesion associated with the surface (Pérez-Miles, Perafán & Santamaría, 2015), which could explain the difference with glass on gradients.

During horizontal displacements, the variations observed for *E*_KIN.x_ were much lower than the variations of *E*_KIN.y_, found in other arachnids (Sensenig & Shultz, 2006; Biancardi et al., 2011). This implies a low energy recovery (Gottschall & Kram, 2006) and therefore a greater energy expenditure by muscles.

According to Moya-Laraño et al. (2008), pendulum mechanics drove the morphological evolution of spiders. In bipeds (mammals and birds), the maximum recovery during horizontal displacement varies between 60–80%, while in quadruped mammals this figure varies between 30–65% (Cavagna, Heglund & Taylor, 1977; Saibene & Minetti, 2003; Griffin, Main & Farley, 2004). However, in arthropods the recovery never exceeds values between 7–19% (Full & Tu, 1990; Full & Tu, 1991; Biancardi et al., 2011; Reinhardt & Blickhan, 2014), and our values (6.9–10.5%) fall in this latter range. The number of locomotor limbs probably influences the inverted pendulum mechanics, as highlighted by the average values of % Cong (47.8–53.0%). Percentage congruity was more similar to that of walking frogs (Ahn, Furrow & Biewener, 2004) than that of grounded running ants (Reinhardt & Blickhan, 2014). In fact, the leg posture of some walking frogs is actually similar to that of tarantulas.

The internal work done by *E. weijenberghi* accounted for 0.2% of the total work, which was lower than the 11% recorded in *G. anthracina* and the 9–15% detected in opilions (Sensenig & Shultz, 2006). Two factors could explain this difference: (i) the speed of the body segments and (ii) the relative mass of the limbs in relation to the body. Any increase of segment speed would imply a higher stride frequency and, consequently, a higher relative speed of locomotion. *W*_INT_ also increases with the speed (Fedak, Heglund & Taylor, 1982; Minetti, 1998; Biancardi et al., 2011).

According to the dynamic similarity hypothesis (Alexander & Jayes, 1983), the Froude number is a dimensionless measure of relative speed and is used to compare the locomotion of individuals and species of different sizes using the following equation:

\begin{eqnarray*}Fr={v}^{2}{g}^{-1}{l}^{-1}, \end{eqnarray*}

where *v* is velocity, *g* is the acceleration due to gravity, and *l* is the characteristic length between the pivot point on the ground and the arc of swing. In ghost crabs, a species with leg segmentation and posture similar to tarantulas, the knee joint (carpopodite-meropodite joint) was used as a reference (Blickhan & Full, 1987). Other authors suggested using the height of the coxa joint from the ground in a normal standing position (Irschick & Jayne, 2000; Biancardi et al., 2011). In order to compare the results obtained for *E. weijenberghi* with *G. anthracina*, we followed the latter suggestion. Even when the absolute locomotion speeds of *E. weijenberghi* were much lower than those of females of *G. anthracina,* the equivalent speeds, which were expressed as number of Froude, were similar.

Moreover, the relative limb masses of males of the *E. weijenberghi* species were higher than those of the female *G. anthracina* spiders (25% vs 13% of body weight). Therefore, a higher relative *W*_INT_ should be expected in the former species yet none of these factors seem to explain the large difference in *W*_INT_ between the two species. Contrary to what was discovered by Minetti, Ardigo & Saibene (1993) and Minetti (1998), the *W*_INT_ decreased with the gradient. We found slight variations in accordance with small decreases in the stride frequency on glass as well as on Teflon. As expected, the *W*_INT_ was proportional to the *F*_S_ on both the horizontal and inclined surfaces (Minetti & Saibene, 1992; Minetti, 1998).

The lower static friction found by Pérez-Miles, Perafán & Santamaría (2015) on Teflon relative to glass probably explains the increased values for the horizontal work we found for the Teflon surfaces. An increment of the mean height of the center of mass was also observed during locomotion on level Teflon surfaces. An upright posture facilitates the contact between adhesive structures and the substrate in geckos (Higham et al., 2015). The hunting spider *Cupiennius salei* adopts a posture on vertical surfaces in order to improve the adhesion (Niederegger & Gorb, 2006). In tarantulas an upright posture could help maintain contact between claw tufts and tarsal claws and the surface because those features are the ones most heavily used during level locomotion (Pérez-Miles, Perafán & Santamaría, 2015).

Contrary to these reports, spiders on inclines assumed a more sprawled position on Teflon than on glass, increasing the supporting area and consequently the stability (Ting, Blickhan & Full, 1994; Weihmann & Blickhan, 2009; Weihmann, 2013). Pérez-Miles, Perafán & Santamaría (2015) observed that in static positive gradients legs I and II interacted with claw tufts while legs IV also touched surfaces with the distal scopulae. Birn-Jeffery & Higham (2014) suggested that animals usually extend their legs or change the pushing mode on gradient surfaces to optimize the force used to overcome gravity (Alexander, 2003). Our results suggest that *E. weijenberghi* needs to increase stability as adhesion diminishes. Despite the implication that adhesion is a costly mechanism of attachment and detachment (Wolff, Nentwig & Gorb, 2013), these costs are probably less than those involved in adopting a crouched posture and using extra muscular force.

**Figure 9:**
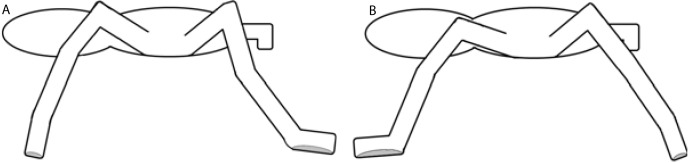
Contact phase of each step. The gray area shows the contact surface of the tarsi. (A) Braking phase: forelimbs produce push adhesion with proximal tarsi, and hind limbs produce pull adhesion with distal claw tufts. (B) Propulsion phase: forelimbs produce pull adhesion with proximal tarsi, and hind limbs produce push adhesion with distal claw tufts.

The adhesion in Theraphosidae is produced by specialized setae located on the ventral face of the distal segments of the limbs. These setae are arranged on claw tufts and tarsal scopulae (Pérez-Miles, 1994). Pérez-Miles, Perafán & Santamaría (2015) and Pérez-Miles et al. (2017) proposed that different adhesive setae produce adhesion when limbs push or pull, according to the part of the limb in question and the orientation of the microtrichiae on the setae. When tarantulas climb, forelimbs pull and adhere to surfaces using distal claw tufts, while hind limbs push and adhere using the scopulae. A similar mechanism has been observed in cockroaches (Clemente & Federle, 2008). The contact phase of each step is composed of a braking phase followed by a second phase of propulsion (Griffin, Main & Farley, 2004; Spagna, Valdivia & Mohan, 2011). We observed that during the braking phase, the forelimbs were in contact with the proximal tarsi (scopulae), producing pushing adhesion, while the hind limbs produced pulling adhesion by contacting surfaces with the distal claw tufts. Conversely, during the propulsion phase, the contacts and adhesion mechanisms worked in reverse, with spiders pushing with their hind limbs and pulling with their forelimbs. During locomotion on a positive gradient, forelimbs should maximize the pulling adhesion by increasing the contact time of the apical tarsi with the substrate, while the hind limbs should increase the contact time with the scopulae to fully utilize the benefits of pushing adhesion (Fig. 9). Adhesive setae have a high adaptive value for the locomotion of spiders in natural environments (Lapinski, Walther & Tschapka, 2015; Pérez-Miles et al., 2017). In fact, the adhesive structures of adult *E. weijenberghi* showed sex-based differences in the static friction angle due to the intense locomotor activity of males (Pérez-Miles, Perafán & Santamaría, 2015).

### Body models

The center of mass of a multi-segmented body varies by changing the relative positions of the segments and is the weighted sum of the centers of mass of each segment in every instance (Zatsiorsky, 2002). Almost all *L*_CoM_ were located close to the geometric center of a cylinder of the same dimension, except for the segment composed of the patella and tibia, probably because the patella is wider than the tibia. When considering variables affected by the movement of the distal body segments, the choice of an adequate multi-segmented model is essential. The two body models of *E. weijenberghi* built in this study have been compared for one experimental condition.

The internal work was indeed the most influenced variable. Reducing the number of leg segments can affect the variation of linear and/or rotational kinetic energy. However, this simplification is necessary to analyze the movements without increasing the number of cameras and, consequently, the processing time (Allard, Blanchi & Aissaoui, 1995).

Finally, it should be noted that, due to methodological limitations in this research, the fovea position was used as a proxy of the true center of mass of the cephalothorax and abdomen (Biancardi et al., 2011). In *Grammostola anthracina* no significant differences were found in the *W*_EXT_, when calculated from the fixed position of a single marker on the fovea versus when it was calculated from the usual weighted position of the centers of mass of all the body segments (Biancardi et al., 2011). In humans it has been shown that, compared to direct dynamic records, *W*_EXT_ could be overestimated by 13% using body models formed by eleven or fourteen segments, and by 16% using a single marker close to the static center of mass (Pavei et al., 2017). In the same investigation it has been shown that the *W*_INT_ was not affected by the different research techniques that involved different calculations to get the *b*_CoM_ (Pavei et al., 2017). The estimations of the vertical, external, and total work differed less than 2% between the two models employed. We consider that both of the models proposed in this research are appropriate to perform a rigorous kinetic analysis in arachnids.

## Conclusions

*E. weijenberghi* locomotion occurs as a variety of different gait patterns. Nevertheless, the most frequently used gait was a walk-trot, similar to the vertebrate trot, but without the flight phase. Time lags and duty factors were consistent between the gaits and provided enough information to differentiate between the roles of the two different limb groups. Indeed, the spider octopedal patterns can be interpreted as the combination of two quadrupeds in series; the anterior mainly steers the movement, while the posterior supports much of the body mass of the arachnid.

The total mechanical work to move one unit of mass a unit distance was largely composed by external work, which in turn was mainly determined by a vertical component dedicated to raising the center of mass against gravity. Horizontal work was disproportionately smaller, determining a very small amount of energy recovery.

With respect to locomotion on inclined surfaces, the changes in mechanical work were in line with those observed in others animals, but these similarities did not extend to values for duty factor, speed, or frequency. While on inclined and slippery surfaces where the internal and horizontal work was greater, spiders adopted postural changes to increase the support area and stability.

##  Supplemental Information

10.7717/peerj.7748/supp-1 Supplemental Information 1 Kinematic data files

## Additional Information and Declarations

### Data Availability

The following information was supplied regarding data availability: Voucher specimens of adult meles Eupalaestrus weijenberghi (Thorell, 1894), Canelones, Salinas, Uruguay (34°45′51.34″S 55°52′49.46″W), Mar. 2–3 2013, F.G. Costa collector, were deposited in Arachnological Collection of the Facultad de Ciencias, Universidad de la República, FCE-MY:1563-1571, 1573-1575.
