# Supplementary material for: Kinematics of male Eupalaestrus weijenberghi (Araneae, Theraphosidae) locomotion on different substrates and inclines
Source: PeerJ. 2019 Sep 26;7:e7748. doi: 10.7717/peerj.7748 (PMC6766366; doi:10.7717/peerj.7748)
Supplement: Supplemental Information 1 [file peerj-07-7748-s001.zip › Silva-Pereyra et al 2019 - Supplementary document - Kinematic data/Silva-Pereyra et al 2019 Suppl Kinematic data.pdf]

Kinematics of male *Eupalaestrus weijenberghi* (Araneae, Theraphosidae) locomotion on different substrates and inclines

Valentina Silva-Pereyra[1], C. Gabriel Fábrica[1], Carlo M. Biancardi[2], Fernando Pérez-Miles[3]

[1] Unidad de Investigación en Biomecánica de la Locomoción Humana, Departamento de Biofísica, Facultad de Medicina, Universidad de la República, Montevideo, Uruguay.

[2] Laboratorio de Biomecánica y Análisis del Movimiento, Departamento de Ciencias Biológicas, Centro Universitario Regional Litoral Norte, Universidad de la República, Paysandú, Uruguay.

[3] Sección Entomología, Facultad de Ciencias, Universidad de la República, Montevideo, Uruguay.

Corresponding author: Valentina Silva-Pereyra, Unidad de Investigación en Biomecánica de la Locomoción Humana, Departamento de Biofísica, Facultad de Medicina, Universidad de la República, Avenida Gral. Flores 2125, 11800 Montevideo, Uruguay, e-mail: vlntnslv@gmail.com

Supplementary document

Kinematic data files

There are twenty kinematics data files, four for each of the five specimens, in agreement with four experimental conditions. The three first numbers in the file name identify the specimen, the letter “v” indicates that the record was over glass and the letter “t” that was

on teflon. The last number indicates the gradient: “0” on level and “12” at 12º of inclination regard to horizontal plane.

We used twenty five markers, one located over the fovea and the rest on the legs. In each leg were located on the tarsus tips, the clear band between metatarsus and tibia, and the middle of patella. With legs numbered from right I to left I clockwise, we list them in the following way: mark 1 correspond to the tarsus of the right leg I, mark 2 to the clear band of the right leg I, and mark 3 to the patella of the same leg; in each leg is form de distal to proximal segment. Thereby, mark 4 correspond to the tarsus of the right leg II, mark 5 to the clear band, mark 6 to the patella and continues like this (Figure 1). There are one file exception in the list markers, the 629v0. The legs was numbered from left I to right I counter-clockwise.

Each text file with .3d extension have 76 columns. The first column indicate the frames (frequency = 0.02), that correspond to two cycles stride plus four frames at the beginning. The second, third and fourth columns correspond to axis x, y, z respectively for marker 1. The fifth, sixth and seventh columns correspond to axis x, y, z for marker 2 and so on. The three last columns: 74, 75 and 76 correspond to the fovea.

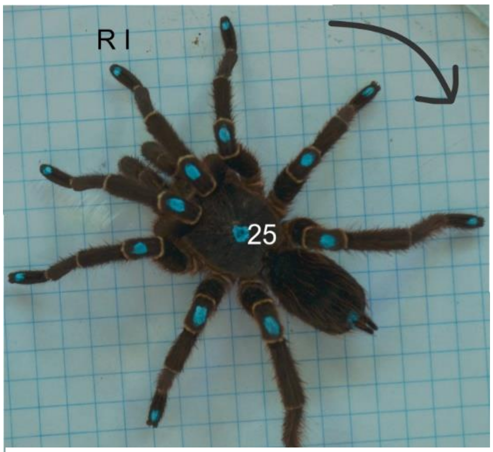

Figure 1. The arrow indicates the direction of leg numbered. RI the first right leg and LIV the left fourth leg. The twenty five indicate the marker over the fovea.

Data matrix of the files:

$$\begin{pmatrix} f_0 & x_{0,1} & y_{0,1} & z_{0,1} & \cdots & x_{0,m} & y_{0,m} & z_{0,m} \\ f_1 & x_{1,1} & y_{1,1} & z_{1,1} & \cdots & x_{1,m} & y_{1,m} & z_{1,m} \\ f_2 & x_{2,1} & y_{2,1} & z_{2,1} & \cdots & x_{2,m} & y_{2,m} & z_{2,m} \\ \vdots & \vdots & \vdots & \vdots & \ddots & \vdots & \vdots & \vdots \\ f_n & x_{n,1} & y_{n,1} & z_{n,1} & \cdots & x_{n,m} & y_{n,m} & z_{n,m} \end{pmatrix}$$

where *n* is a sequential number identifying the row (frame), and *m* is the number of the body marker (1 to 25).

Orthogonal axes were defined as follows: the direction of x-axis agreed with the main displacement direction, z-axis agreed with height with respect to the ground and y-axis was determinate by the right-hand rule.

The files recorded over teflon have “NAN” in the columns that correspond to the clear bands because we did not digitized that mark on this videos but the others marks conserve the position in the matrix.
